# Earthworms, Rice Straw, and Plant Interactions Change the Organic Connections in Soil and Promote the Decontamination of Cadmium in Soil

**DOI:** 10.3390/ijerph15112398

**Published:** 2018-10-29

**Authors:** Ali Mohamed Elyamine, Mohamed G. Moussa, Marwa A. Ismael, Jia Wei, Yuanyuan Zhao, Yupeng Wu, Chengxiao Hu

**Affiliations:** 1 Key Laboratory of Arable Land Conservation (Middle and Lower Reaches of Yangtze River), Ministry of Agriculture, Research Center of Micro-Elements, College of Resource and Environment, Huazhong Agricultural University, Wuhan 430070, China; elyoh@hotmail.fr (A.M.E.); MohamedGomaa_Ali@agr.asu.edu.eg (M.G.M.); maf02@fayoum.edu.eg (M.A.I.); jiawei7@webmail.hzau.edu.cn (J.W.); zyy235813@163.com (Y.Z.); wyp19851205@126.com (Y.W.); 2 Hubei Provincial Engineering Laboratory for New Fertilizers, Huazhong Agricultural University, Wuhan 430070, China; 3 Department of Life Science, Faculty of Science and Technology, University of Comoros, Moroni 269, Comoros; 4 Botany Department, Faculty of Science, Fayoum University, Fayoum 63514, Egypt

**Keywords:** cadmium, earthworm, rice straw, bioavailability, bioaccumulation, Cd fractionation

## Abstract

The joint effects of earthworms and crop straw on toxic metal speciation are not clear, and very limited information is available regarding the effects of their interaction on Cd mobility in Cd contaminated soil or in remediation processes involving plants. This study evaluated their impacts on Cd mobile form changes in soil and their effects on Cd uptake by plants. Treatments included both planted and unplanted-Cd-contaminated soil with or without rice straw and/or earthworms. The results revealed that earthworms, rice straw, and plant interactions change the Cd mobile forms in soil. The order of Cd concentration of different chemical forms was as follows: exchangeable > residual > bound to Fe-Mn oxide > bound to organic matter for earthworms, and exchangeable > bound to organic matter > residual > bound to Fe-Mn oxide for rice straw treatment, with a recovery rate of 96 ± 3%. The accumulation of Cd in plants increased in the presence of earthworms and decreased in the presence of rice straw. FT-IR spectra indicated that the degradation of rice straw increases C–O, C–O–H, C–H, and O–H functional groups which could complex with Cd ions. These findings highlighted that earthworms’ activities and crop straw can modify soil properties and structure and promote the remediation of heavy metal. This study suggests that the ecological context of remediation instead of being limiting on soil-earthworms-plant interaction, should integrate the natural resources forsaken which can provide a positive influence on both plant health and the remediation of heavy metal in contaminated soil.

## 1. Introduction

The contamination of soil with heavy metals, apart from naturally occurring deposits, is strongly related to anthropogenic activities, particularly the application of pesticides and inorganic fertilizers to agricultural soils [1,2], industries including metal-plating, mining, tannery, petrochemical, textile, battery and fertilizer production [3], and the discharge of wastes containing heavy metals [4]. Cadmium (Cd) is one of the most toxic pollutants because of its non-degradability, persistence in nature, and high toxicity to plants, soil organisms and human beings [5]. It was reported to negatively affect plant physiological growth [6], soil organism biomass [7], and at a given concentration, it may affect humans’ health through the food chain [8].

Diverse techniques ranging from traditional ones (chemical or thermal) to green technologies are used to clean up heavy metals that have contaminated the environment. Bioremediation and phytoremediation using the natural ability of plants, fungi, bacteria or algae to clean up organic or inorganic pollutants are widely accepted because they are environmentally friendly and they are economic [9,10]. The efficiency of the remediation process depends on the plant species, the availability of the metals, organic components, and biological and chemical properties of the soil which are key factors related and connected in such a way that the change of any of them could affect the available form of metal and its accessibility to plants [11,12]. Soil organic matter in some cases may be a limiting factor which can affect the total and mobile forms of heavy metals and decrease their accumulation in a bioremediating organism [13]. Therefore, its degradation could re-establish the active form of metals and therefore increase its bioavailability in soil and their accumulation in plants and in others bioremediating organism.

Earthworms defined as “soil engineers”, by Charles Darwin are bio-indicators of contaminated-soil, ameliorating the soil structure by increasing soil airing [14,15] and are implicated in the conveyance of soil microorganisms [16]. Earthworms feed on the litter deposited at the soil surface, soil organic matter and soil [14]. Organic and minerals compounds are mixed in the gut and rejected in form of casts on the soil surface or along burrows [15]. The burrows and casts produced by earthworms are reported to enhance the connection between microorganisms and pollutants [16]. Earthworms were also shown to support phytoremediation of heavy metals by changing the soil pH and altering the structural community of soil bacteria [15]. Several reports have emphasized the role of earthworms on metal speciation and their ecological context including soil-plant interaction is widely investigated [16]. However, information about their interactions including earthworms-soil-plant and crop straw on the remediation of heavy metals is limited.

Rice straw as a heterogeneous material could be used for diverse uses; as an organic fertilizer, it can improve the crop quality and biomass and also accelerate plant growth by providing the necessary nutrients [17]. Nowadays, due to the lignocellulose composition of its fibers and the fast reduction of fossil fuel-reserves as well as the negative environmental consequences resulting from the inflated utilisation of fossil fuels, rice straw was proved to be an excellent candidate for biogas production, either by pyrolysis processes or by other chemical treatments [18,19,20]. In addition, due to its multitude of functional groups, rice straw is an effective tool as a biosorbent for reducing heavy metals in aquatic environments [21,22]. However, applications of rice straw to remove heavy metals in agricultural soil are rare. To the best of our knowledge, the effect(s) of the interaction of earthworms and rice straw on Cd availability and mobility in remediation processes involving plant (root) systems in contaminated agricultural soil and their combined effects on toxic metal speciation are not clear. Since earthworms and rice straw may change Cd bioavailability in the soil, their influence on metal speciation might be particularly essential in contaminated soils [23]. This study was thus carried out to explore how earthworms and rice straw influence Cd total and active forms in the soil. The study supports the hypothesis that the interaction of rice straw and earthworms could not only change the organic connections in soil, and affect Cd mobility and bioavailability, but also will increase the number of functional groups which might immobilize Cd in soil. It aims to: (i) examine the effect of the inoculation of earthworms and rice straw on Cd bioavailability and mobility; and (ii) evaluate their efficiency in phytoremediation processes.

## 2. Materials and Methods

### 2.1. Soil Properties

Soil was collected from the test field at Huazhong Agricultural University (HZAU) (30°28′26″ N, 114°20′51″ E). The upper litter was removed, and the soil from the top layer (0–20 cm) was collected. The soil samples were transferred to the greenhouse of the Micro-element Research Center at HZAU, for grinding and sieving. The soil used presented the following properties: pH (soil: H_2_O 1:2.5) 7.6; organic matter 1.31%; NH_4_Cl exchangeable K, 127.99 mg/kg; total nitrogen N 0.17%; Olsen-P of 39.69 mg/kg; CEC 11.47 cmol^+^/kg; and Ca, 2288.2 mg/kg.

### 2.2. Earthworms and Rice Straw

Earthworm species (*Aporrectodea caliginosa* and *Eisenia fetida*) belonging to two different ecotypes (endogeic and epigeic, respectively) were selected for this study. A litter-dwelling *E. fetida* was chosen for its fast growth, rapid productivity and its organic waste conversion efficiency [24], while the horizontally burrowing mineral soil feeder *A. caliginosa* was selected for its ability to transfer nutrients or chemicals elements within a compartment of an ecosystem or between different compartments [25]. Both earthworm species were chosen and selected in the earthworms breeding site at HZAU. A sufficient quantity of earthworms (for different species) was initially purchased from a commercial source and transferred to the greenhouse where a controlled earthworm breeding site containing soil filled with household waste (cabbage waste, carrot peelings, banana waste) was installed. The worms used in the present study were selected after 3 months of reproduction in this mentioned site, washed in deionized water, placed on wet filter paper and maintained in the darkness at 20 ± 2 °C for one night in the laboratory [26] before placing them in the surface of the corresponding experimental pot. Rice straw of *Oryza sativa* (Taichung Native-1 variety) was collected at the agricultural field of HZAU, transferred to the laboratory where it was dried at 60 °C and ground to 2 mm.

### 2.3. Experiment Design

Five kg of air-dried soil was placed in ceramic pots (22 × 21 cm). Cadmium concentrations of 0.5, 1 and 3 mg/kg of dry soil were applied as cadmium chloride solutions (CdCl_2_, 98%, purity) [27]. This solution was poured on the soil surface and the soil matrix was thoroughly mixed and incubated at 20 ± 1 °C [26] for three months. Throughout the incubation time, the soil moisture content was scrutinized each week by using a mobile Time Domain Reflectometry (TRD-100, Campbell Scientific (Beijing) Co., Ltd., Beijing, China) and maintained by watering with DI water if needed.

**Figure 1:**
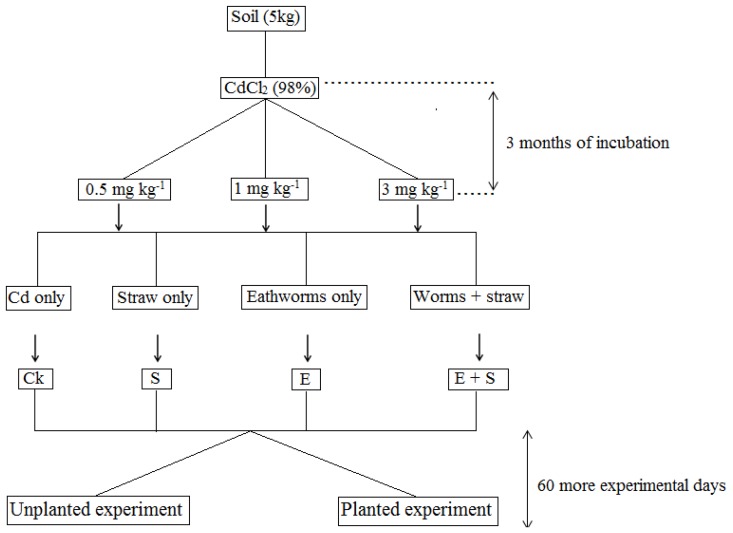
Summary of experimental design with all different treatments.

After 3 months of incubation, the prepared and inoculated pots with Cd were used for the experiment and they were either left unplanted or planted for 60 more experimental days. Each experimental type has four treatments with three replicates including the control treatment (Ck) with neither rice straw nor earthworms; S treatment with rice straw only; E treatment with earthworms only and E+S treatment with both earthworms and rice straw (Figure 1). Ground straw (to 2 mm particle size, 500 g/kg soil) was applied on the soil surface of the S and E + S treatments; the soil matrix was thoroughly mixed and then moistened with deionized water up to 70% of the water holding capacity. For each species of earthworms, 10 adults and healthy individuals worms with similar fresh weight (1.84 ± 0.06 g and 3.03 ± 0.04 g for *E. fetida* and *A. caliginosa,* respectively) were randomly selected, regrouped and rinsed with deionized water and placed on moist paper for 24 h to void the gut content before placement on the corresponding soil surface of E and E + S treatments. The initial body weight of earthworms was measured immediately before placing the animals on the pot experiment. After 60-days, the final body weight was taken for body weight evaluation. The mortality of earthworms in the pre-incubation treatment was monitored every 5 days. Earthworms were considered dead when they did not show any response on probing.

In the planted experiment, oat (*Avena sativa*) and sunflower (*Helianthus annuus*) seedlings (five for each species) were planted in separate prepared pots and placed in the greenhouse. To avoiding any mixing-up of earthworms between the treatments, pots were kept separately. During the whole experimental period of 60 days, plants in pots were watered daily and monitored weekly. At the 60th day, soil sample was taken to test different studied parameters. Roots of sunflower and oat plant were separated from their shoot, and cleaned with water from piped supply to remove adhering soils before being washed with DI water. Root and shoot fresh weights were taken before dried at 60 °C for 7 days. The root or shoot biomass were registered as the dried mass.

### 2.4. Analytical Test

Soil pH [28] and Dissolved Organic Carbon (DOC) [28] was determined as per the American Society for Testing Materials (ASTM) standards. To conduct total Cd in plant tissue analysis, double acid digestion of the sample was applied as previously described [29]. Briefly, approximately 0.2 g of grinded plant sample was digested using 10 mL of HNO_3_/HClO_4_ (4:1) mixture at 200 °C. The digested solution was cooled and diluted to 50 mL using deionized water. The diluted solution was filtered and Cd concentrations were measured by Atomic Absorption Spectrophotometry (AAS) (Z-2000, HITACHI, Tokyo, Japan).

At the 60th days, earthworms were sorted from each pot, washed in deionized water, and placed on wet filter paper to allow depuration for 48 h. Earthworms were then washed again, freeze-dried and ground. A sub-sample of 500 mg was used for Cd analysis. Earthworm sample was digested with 6 mL of HNO_3_/H_2_O_2_ mixture (5:1) on a hot plate at 150 °C for 2 h [25]. The digested solution was evaporated to 1 mL and 1% HNO_3_ was added to adjust the volume to 25 mL. The diluted solution was filtered and Cd concentrations were measured by AAS (Z-2000, HITACHI, Tokyo, Japan).

**Figure 2:**
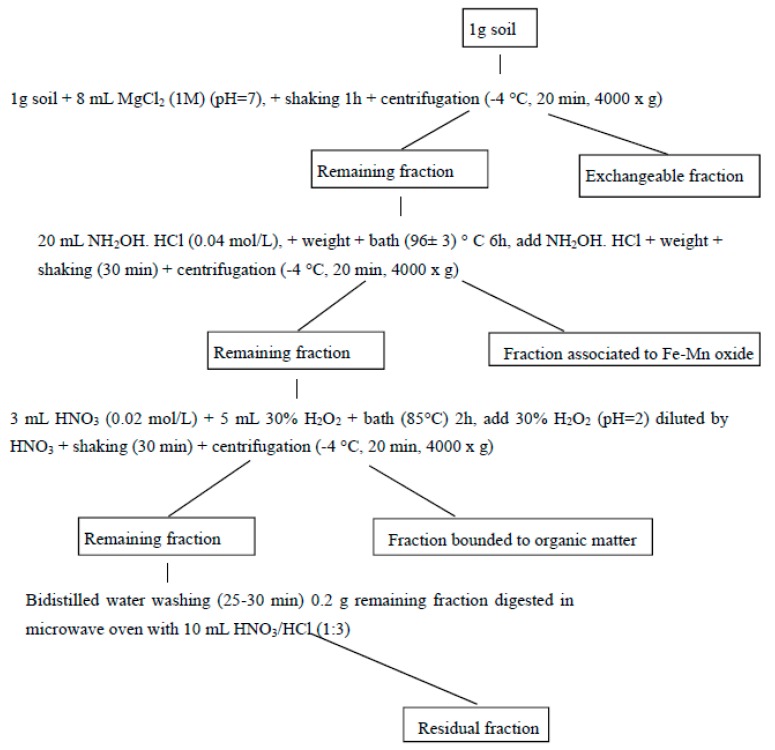
Operating conditions used in Cd sequential extraction procedure.

In order to depict the influence of both earthworms and rice straw on Cd mobility and availability, Cd in each extracted fractions was estimated by adopting the sequential method inspired in [30] with slight modifications. Sequential extraction was performed according to the Figure 2. Briefly, 1 M magnesium chloride (pH 7) was used to extract the exchangeable Cd fraction; 0.04 mol/L hydroxylamine hydrochloride (pH 2) to extract the fraction bound to Fe/Mn oxide; 30% m/v H_2_O_2_ and 0.02 mol/L nitric acid (pH 2) to extract the fraction bound to organic matter and nitric acid/hydrochloride acid to extract the residual fraction of the Cd. Cd concentration in each soil sample and for each fraction was determined in four repetitions and the solution was subjected to AAS to analyze Cd concentration.

To determine how far the method used could permit to extract the different Cd forms in the different samples, the accuracy and analysis quality of Cd measurements was checked by using certified standard material GBW07405 (GSS-5) with a well-known Cd concentration, purchased from National Central of Standard Materials in China. Cd concentration in the reference material was determined in the same manner as in any other sample. Ainsi the recovery rate calculated following the formula below, for the sequential extraction was 96 ± 3%:

Recovery (%)=∑(E+FeMn+OM+R)×100/Total Cd in soil

where E: exchangeable Cd, FeMn: Cd bound to Fe-Mn oxide, OM: Cd bound to organic matter and R: residual Cd in soil.

### 2.5. Bioconcentration Factor, Translocation Factor and Kinetics Parameters k_1_ and k_2_

Bioconcentration factor (BCF) and translocation factor (TF) for plants were respectively calculated as the ratio of the Cd content in the plant to that in the soil and the ratio of the Cd content in the shoot to that in the root.

To depict the impact of earthworms on Cd availability and mobility, a distinct accumulation-depuration experiment was carried out in parallel for 6 days in which the activities were monitored by 0, 2, 4 and 6 day. BCF, the uptake rate constant (*k*_1_) and elimination rate constant (*k*_2_) were determined as described by [31] and the famished earthworms were kept at −28 °C for further analysis. Elimination constant rate (*k*_2_) was determine as the concentration eliminated per day and the uptake constant rate (*k*_1_) was calculated by using the formula described by [32]: *k*_1_ = BCF × *k*_2_.

### 2.6. Fourier Transform Infrared Spectra of Rice Straw

FT-IR analysis of rice straw was performed as described in [21]. In brief, the freeze-dried rice straw sample (10 mg) was mixed with KBr (100 mg), ground, homogenized and pressed to reduce light scatter. Spectra were obtained by scanning the sample from 4000 to 400 cm^−1^ at 1 cm^−1^ resolution using Nicolet FTIR iS10 (ThermoFisher Scientific, Co., Ltd., Beijing, China).

### 2.7. Statistical Analysis 

All data were subjected to the Analysis of Variance (ANOVA) using Statistical Package for Social Science (SPSS. 20, IBM Company, Chicago, IL, USA) statistical software, One and two-way ANOVA followed by Least significant Different (LSD) with 95% confidence level were performed to assess the differences among means and multiple stepwise. Linear regression was used to find out the correlation between the concentration of pollutant and different parameters. Different graphs were performed using SigmaPlot 12.0 software (Systat Software Inc., Chicago, IL, USA).

## 3. Results

### 3.1. Earthworm Survival and Body Weight Variation

In order to assess if there was a change in mortality of earthworms after 60 days of Cd exposure, ANOVA, *p =* 0.05 was performed. The mortality rates in different Cd-contaminated soils presented no differences compared to that of the control (data not shown). In addition, whatever the Cd concentration applied in soil, the cropping of either sunflower or oat plant did not show any effect on both earthworm species mortality. However, considering rice straw as a food for earthworms, it addition significantly reduced both earthworms mortality rates (*p* = 0.024).

Earthworms’ weight was related to three factors: food supply/species feeding, the presence of the plants, and soil contamination. Comparing the effects of these three factors individually, body weight of both earthworms after treatment with rice straw (E + S) was found to be significantly different (ANOVA, *p* = 0.05) from that in the pots without rice straw (Table 1). Thus, a separate two-way analysis of variance test was performed between treatment with or without food supply by considering the other two factors. The factor “rice straw supply” (*p =* 0.041, *r*^2^ = 0.0830 and *p* = 0.037, *r*^2^ = 0.0881 respectively for *E. fetida* and *A. caliginosa*) and “presence of plant” (*p* = 0.027, *r*^2^ = 0.065 and *p* = 0.038, *r*^2^ = 0.0681) showed significant and positive interaction with earthworms body weight. The body mass of *E. fetida* and *A. caliginosa* in food supply treatment was respectively increased by about 19% and 17%; while in cropping pots, the increase was about 9% and 7% respectively for *E. fetida* and *A. caliginosa*. Though there was positive interaction between plant and earthworms’ body weight, the individual influence of sunflower (E_1_) was significantly higher compared to that of oat (E_2_).

### 3.2. Soil Physical and Chemical Analysis

Soil samples were collected from the four different treatments, including planted and unplanted soil for analyzing pH, DOC, cation exchange capacity (CEC) and organic matter content (Table 2) to investigate their effect on Cd availability and mobility.

Soil pH varied from 7.28 to 6.04 and from 7.4 to 5.91 in unplanted and planted soil, respectively. No significant differences in soil pH values were observed among planted and unplanted soil. However, the inoculation of earthworms either in separate or dual treatment significantly decreased soil pH in both soils. No significant effect on soil pH was observed in rice straw treatment.

DOC content was found to be significantly different among both planted and unplanted soils. Soil sample from unplanted treatment showed low DOC content compared to that of the planted soil. Additionally, the inoculation of rice straw significantly (ANOVA, *p* < 0.05) increased DOC compared to the other three treatments.

Earthworms’ addition increased CEC by about 3.91 units; while the inoculation of straw decreased it by about 1.25 units. Although the inoculation of earthworms increased organic matter content, S treatment presented the highest content (*p* < 0.001). Two-way ANOVA disclosed that Cd concentration in soil had a significant effect on CEC and OM concentration. 

### 3.3. Cd Concentration

#### 3.3.1. Cd Accumulation in Earthworms

The mean concentrations of Cd for both earthworms’ species were measured and reported in Table 3*.* Cd concentration in both earthworm species was significantly different among the different treatments. Thereby, to understand the factors influencing Cd accumulation in earthworms, a separate two-way analysis of variance was performed by considering “rice straw supplied”, “Cd concentration” and “plant” factors. It was revealed that “rice straw” and “plant” factors showed significant (*p* = 0.013 and *p* = 0.018 respectively) interaction with Cd concentration in both earthworms tissues. The inoculation of rice straw resulted in a significant (*p* < 0.05) increase of Cd accumulation in both earthworms species at all Cd concentration. However, the presence of both plants (E_1_ and E_2_) significantly decreased Cd concentrations in both earthworm species. “Cd concentration variation” factor did not manifest any significant interaction, despite the fact that Cd concentration in both earthworms species tissues clearly increased with exposure to raised soil Cd concentration. 

BCF, the uptake rate (*k*_1_) and elimination rate (*k*_2_) constants in earthworm body tissues are presented in Table 4. BCF in both earthworm species decreased gradually with the increase of Cd concentration in soil. No significant differences were observed in the Cd uptake rate constant (*k*_1_) in both earthworm species within the exposure level, however, with respect to each earthworm’s specie, the corresponding parameters values increased with elevated exposure level. Contrary to uptake constant, the elimination rate (*k*_2_) constant of both earthworm species was significantly different.

#### 3.3.2. Cd Accumulation in Plant Tissues 

**Figure 3:**
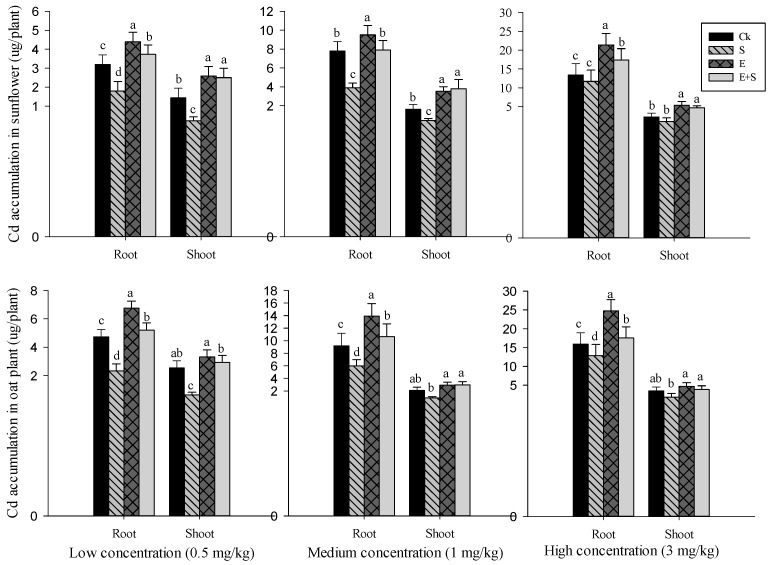
Effect of earthworms (E) and rice straw (S) on sunflower and oat plant root and shoot Cd accumulation under different Cd concentrations (low, 0.5 (1) medium, 1 (2) and high, 3 (3) mg/kg Cd) treatments after 60 exposure days. Different colors (Black, grey, tan…) vertical band present the mean of three replicate and were compared by Tukey’s tests. The lowercase letters in the same group (four vertical bands) indicates significant difference at *p* < 0.05.

The mean of Cd concentrations in sunflower and oat plants with and without rice straw and earthworms are plotted in Figure 3. Earthworms either in separate (E) or dual (E + S) treatment significantly increased the Cd concentration in roots and shoots of both plants. In contrast, the inoculation of rice straw (S) significantly (*p* < 0.05) decreased Cd accumulation in both roots and shoots of sunflower and oat plants. Though Cd concentration in oat plant tissues seems to be slightly higher compared to that of sunflower, the difference was not significant. When the Cd concentration in plant tissues was compared to the available Cd concentration in different soils, no correlation was noted for both plants. However, as earthworms significantly increased plant biomass (result not shown) and Cd concentration in plants tissues, Cd accumulation (plant dry weight × Cd concentration) was increased in sunflower and oat root by 28% and 18% and shoot by 37% and 20%, respectively, compared to the control. In contrast, compared with Ck, the inoculation of rice straw significantly decreased Cd concentration in sunflower and in oat root and shoot by about 11% and 32% and by 26% and 35%, respectively.

BCF for both plants in control treatment at low Cd concentration was greater than 1 (Table 5). However, it gradually decreased with an increase in Cd concentration in soil. S treatment significantly decreased both BCF and TF in both plants. Nevertheless, the inoculation of earthworms either in separate (E) or combined (E + S) treatment significantly increased both BCF and TF in both plants.

#### 3.3.3. Cd Bioavailability and Mobility 

**Figure 4:**
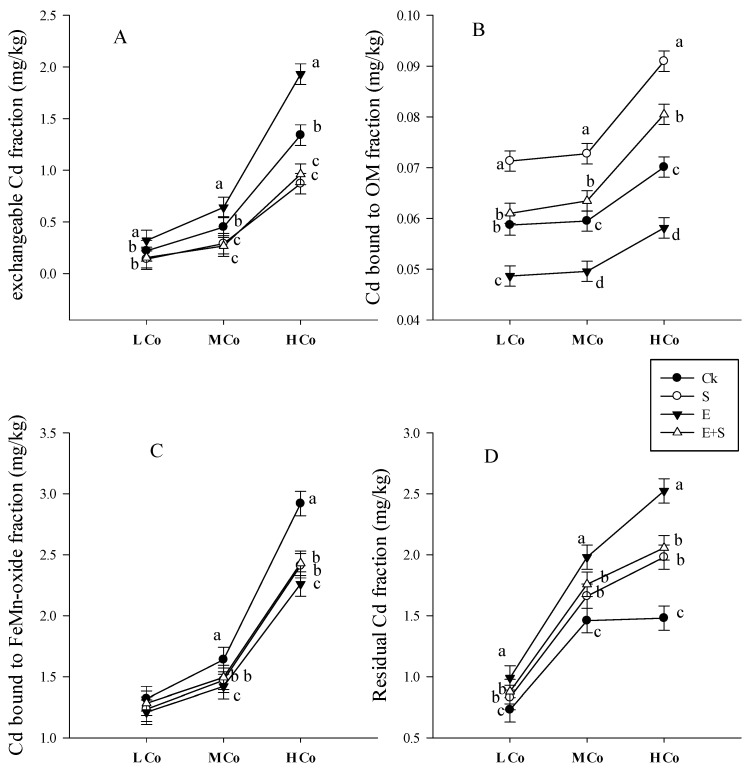
Effect of earthworms (E) and rice straw (S) on Cd fraction extracted in unplanted different soil divided into exchangeable (**A**), bound to organic matter (**B**), bound to iron-manganese oxide (**C**) and residual fraction (**D**) under different Cd concentration (L Co (low concentration), 0.5 M Co (medium concentration), 1 and H Co (high concentration), 3 mg/kg Cd) treatments after 60 exposure days. Different colored points (Black, grey, tan…) present the mean of three replicate and were compared by Tukey’s tests. The lowercase letters in the same group (4 points) indicates significant difference at *p* < 0.001.

Both planted and unplanted soils were subjected to the sequential extraction in which Cd was divided into exchangeable, bound to organic matter, bound to iron-manganese oxide and residual fraction. The point to be noted was that in planted soil, the availability of Cd significantly decreased especially in S and E treatments. Thus, to have a clear understanding of how both rice straw and earthworms affect the fractional distribution of Cd in the soil, “plant cropping” factor was excluded from the analysis and only the unplanted soil was considered. The addition of earthworms significantly affected the fractionation of Cd by increasing exchangeable Cd (Figure 4).

However, it resulted in a decrease in the fraction of Cd bound to organic matter and Fe–Mn oxide by increasing the residual Cd fraction. The inoculation of rice straw significantly decreased the availability of Cd by increasing the organic content and probably by transforming inorganic Cd into organic form. In general, the order of Cd concentration of different chemical forms was R > Fe–Mn oxide > OM for earthworms, and OM > R > Fe–Mn oxide for rice straw treatment with a recovery rate of 96 ± 3%. 

### 3.4. Fourier Transform Infrared Spectra of Rice Straw

**Figure 5:**
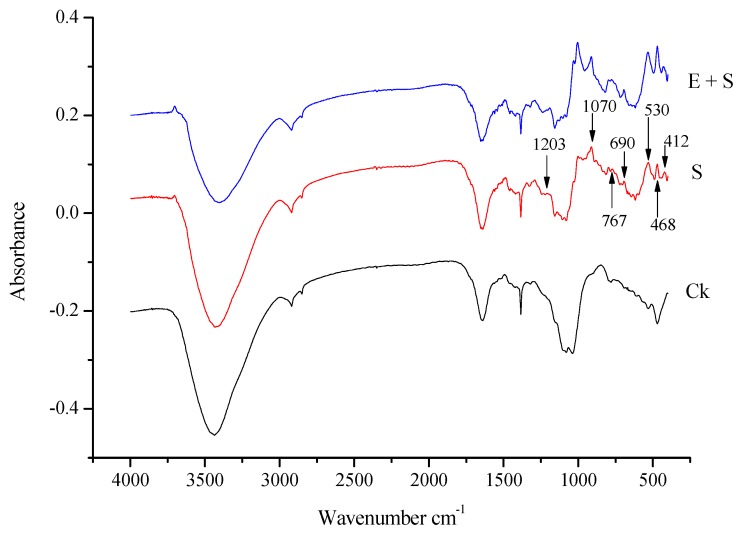
FT-IR in 4000–400 cm^−1^ spectra region of rice straw. Ck indicates the spectra of rice straw untreated (natural); S, that of RS in soil and E + S the spectra of RS in soil inoculated with earthworms.

To understand the change of the functional groups of rice straw in soil (S) and in soil inoculated with earhworms (SW), the FT-IR absorption spectra of rice straw (Figure 5) were performed. Basically, rice straw is constituted by cellulose, hemicellulose, lignin, extractives, water and silica. The characteristic absorption bands and different functional groups of rice straw corresponding to different peaks in Figure 5 are listed in Table 6. Although the three spectra of Ck, S and E + S present a very similar profile from 1232–3440 cm^−1^, there were displacements between the three rice straw samples. The main changes of absorption bands were observed from 1070 to 400 cm^−^^1^ corresponding to C–O, C–O–H, C–H and O–H functional groups.

## 4. Discussion 

In this study, we investigated the impacts of earthworms and rice straw on cadmium availability and mobility. We found that rice straw could be used as a food source for earthworms and could enhance earthworms’ body weight. Additionally, both earthworm species and rice straw not only improved soil physical and chemical properties, but also changed the Cd chemical form which altered Cd bioavailability and bioaccessibility to the plant.

### 4.1. Earthworm Survival and Body Weight Variation

Throughout the experimental period, the high survival rate observed for both earthworms species suggests that the experimental conditions were acceptable in terms of providing a vital environment and suitable media for earthworm survival. Additionally, the low range of Cd contamination applied in the present study may contribute to the observed result. This confirmed the previous report showed that both species were less sensitive to metal stress [33].

The inoculation of rice straw significantly increased the body weight of both earthworm species compared to their initial body weight (Table 1). Generally, the loss of body weight in a contaminated-soil is related to the earthworms’ strategy once exposed into stress conditions. Indeed, once exposed in a polluted environment, earthworms are able to attenuate the toxins and its resulting effects by regulating their internal biochemical activities [34,35]. As the result of this study is not consistent with the general strategy of earthworms under toxin stress, our findings support the hypothesis that the variation in body weight of both worms species was mostly related to food supply rather than metal contamination. This could be explained by the low Cd availability due to the interaction between Cd and different soil materials [36]. Soil properties were reported to affect metal bioavailability and change their impact on earthworms’ physiology [37]. However, rice straw decomposition could increase soil microbial content which in return constitutes nourishment for earthworms.

The body weight of both worm species was shown to increase in sunflower planted pots. This may be explained by the direct effect of earthworms to feed on roots, especially dead roots of plants [38] and indirectly by the enrichment of soil in microbes and the decrease of Cd in soil due to plant root exudate and metal uptake by the plant. Sunflower through the root exudates releases substances which support the development of microbial colonies by providing 10 to 20% of the sources produced by photosynthesis [39], and produce enzymes such as oxidase and peroxidase, and organic acids such as malate, oxalate and citrate, which are able to complex with metals and facilitate their rhizo-absorption [40].

### 4.2. Cd Accumulation

#### 4.2.1. Earthworms

Earthworms are recognized to take up and accumulate metals by two pathways: (1) by direct dermal contact with metals in soil and (2) intestinal by the ingestion of bulk contaminated soil [41,42]. The metals distribution and their availability determine their toxicity to any living organism in soil [43]. At a given concentration when the metal uptake by an individual worm reaches a distinct threshold level, the worms actively eliminate the excess in their cast. This implies that the elimination process is based on the transport of Cd into internal storage compartments [44]. BCF for both worm species gradually decreased with the increase of Cd concentration in soil (Table 4). This supports the observation that, at low Cd concentration, earthworms absorb and accumulate Cd in their tissues. In contrast, at higher concentrations, earthworms could absorb Cd but not to accumulate it, and thus, worms eliminate Cd into their cast [44]. When the bulk soils are taken up by worms, it passes through their guts; enzyme, surfactant and the mucus secreted into the content changes the metal form [45]. This could explain the increase of exchangeable Cd fraction (Figure 4A) and why the elimination rate constant *k*_2_ in both earthworms was different from the uptake rate constant *k*_1_ (Table 4). The presence of plants reduced the accumulation of Cd in earthworms. This was probably due to the fact that plants accumulated part of Cd.

#### 4.2.2. Plants

The accumulation of pollutants in plants to a varying degree depends on several conditions such as plant species, the type and properties of soil, nature of the pollutant and its available form [46]. In addition, their transfer from root to shoot may be attributed to plant capacity to produce metal transporters such us Cyclic-Nucleotide-Gated-Channel [47], protein-ligand, phytochelatins and other metallothioneins [48]. Both sunflower and oat plants respectively accumulated a consequent amount of Cd ranging from 5.8–11.53 µg/plant and 7.05–15.12 µg/plant in their respective roots and 0.57–3.88 µg/plant and 1.11–3.66 µg/plant in their respective shoots. In addition, their respective BCF in control treatment at low Cd concentration was greater than 1 (Table 5) indicating that sunflower and oat are Cd accumulator plants. This, therefore, suggested that both plants can enable Cd extraction from contaminated soil despite the few studies involved their use in phytoremediation process, because they are not considered as metal tolerant plant species. However, sunflower is a great candidate for biodiesel production [49,50] and oat plant in plant physio-chemistry studies [51,52]. In the present study, Cd concentration in oat plant tissues was shown to be slightly higher compared to that of sunflower. The difference between these two plants to accumulate Cd could be related to the difference of mechanisms in metal uptake. Based on plant accumulation, Chirakkara et al. [53] and Pilon-Smits [54] reported that a possible explanation of the difference between both plants was due to the membrane transporter proteins. Indeed, these proteins responsible for the absorption of nutrients from the soil and their transport into the plant could thereby be involved in metals uptake and transfer. Thus, we can conclude that the uptake of metals by plants is not only related to its dispersion in soil but also by the exposure trails and plants physiology, which the detailed mechanism needs further investigation. 

### 4.3. Earthworms Change Chemical Form of Cd

**Figure 6:**
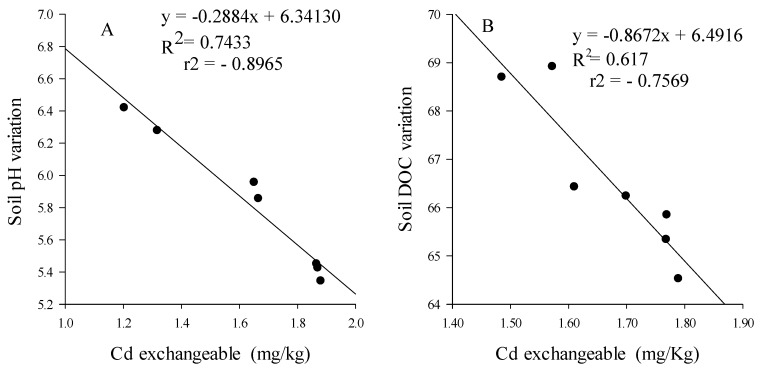
Linear regression performed to set up the correlation between Cd availability and soil pH (**A**) and soil DOC (**B**) variation.

Earthworms increased Cd availability (Figure 4) and decreased the soil pH (Table 2). Thus, linear regression analysis was performed to set up whether there is a correlation between Cd availability and soil pH variation. Soil pH variation was found to have significant and negative correlation (*r*^2^ = −0.89, *p =* 0.039) with Cd availability (Figure 6A). This result is consistent with most of previous studies, which reported that soil properties can affect metal bioavailability and change their impact in soil organism and plant [46]. Although the mechanisms by which earthworms decrease soil pH are still unclear, the variation of pH most likely due to the release of humic substances and fulvic acid during the decomposition process induced by earthworm [55]. Yu et al. [56] reported that the mobility of metal increased when soil pH decrease. Decreasing soil pH could enhances the cation mobility in soil pore water as result of the replacement of H^+^ by the exchangeable cations from soil [57] and that of the solubility of metal bounded with soil different materials [58].

### 4.4. Rice Straw Change Chemical Form of Cd and Sequestrate It in Soil 

**Figure 7:**
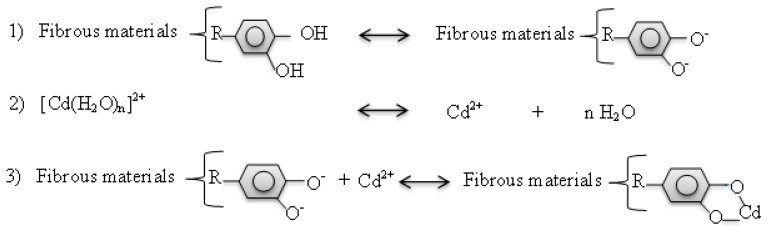
Proposed mechanism for rice straw absorption and sequestration of Cd in soil.

The application of crops straw based on the mechanism of precipitation and adsorption (including electrostatic and specific adsorption) is widely used to decontaminate water polluted by heavy metals [59]. Rice straw, through its chemical structure and composition may bind with heavy metals and immobilize it in soil. The inoculation of rice straw significantly increased DOC content (Table 2), the residual fraction of Cd, Cd bound to organic matter and decreased Cd availability (Figure 4). We thereby performed a linear regression analysis test to investigate the correlation between Cd availability and soil DOC content. It was revealed that DOC content significantly and negatively correlate (*r*^2^ = −0.75, *p =* 0.41) with Cd availability (Figure 6B). This result is consistent with that found by [60,61] which have shown that the mineralization and the humification of soil organic matter significantly changes the metal behavior and reduces the exchangeable fraction of the initial given heavy metal, their water-soluble ions and converts them into a residual fraction. Indeed, soil organic matter transformation occurs both in the mineralisation and humification processes and the direction of these processes is governed by the composition of the humic substances [61]. Humification of organic matter tended to promote the formation of fulvic acids which was reported to having a high capacity for divalent cation metal complexation [62]. A high proportion of humified organic matter can decrease the bioavailability of heavy metals in soil by adsorption and by forming stable complexes with humic substances [61]. This OM can re-distribute heavy metals from soluble and exchangeable forms (extractable metal) to fractions associated with OM or carbonates and the residual fraction. However, the binding affinities of Cd with soil organic matter were reported to be variable and to depend on its functional group composition and structure [21]. FTIR spectra of rice straw revealed that the main changes of absorption bands were observed from 1070 to 400 cm^−^^1^ corresponding to C–O, C–O–H, C–H and O–H functional groups (Figure 5). The increase of these functional groups suggested the degradation of rice straw. Huang et al. [22] reported that the sequence of binding affinities of Cd with rice straw-derived DOM was C=O > OH deformation > COO-symmetric and asymmetric > C=C stretching aromatic. Thus, we can speculate that the decomposition of rice straw in soil led to an increased number of functional groups which can complex with Cd cation. However, the principal mechanism by which rice straw immobilized Cd in soil is still unclear. Meanwhile, based on the ion exchange process and the chelation of Cd with the organic functional group composing the fibrous of rice straw and relying on the scheme proposed by Rocha et al. [21] a possible mechanism for rice straw to absorb and immobilize Cd in the soil is proposed in 3 steps (Figure 7). Step 1: the organic material composing the rice straw fibrous loses two protons per mol of divalent cation. Step 2: the hydrated Cd loses its hydration water and in Step 3 the organic material of rice straw coordinates to take up the non-solvated Cd. 

Thereby, rice straw as a heterogeneous material formed by several types of sites could interact with Cd and decrease its available concentration. 

### 4.5. Earthworms, Rice Straw and Plants Interaction 

The presence of earthworms increased the Cd concentration in both sunflower and oat plants. This result was consistent with that of Kaur et al. [63], who reported that earthworms’ activities increased Cd uptake in *Brassica juncea* L. As earthworms are known to transport soil microorganisms and enhance the connection between microorganisms and pollutants [16], we can assume that the effect of earthworms on metal uptake by plants result in part from their impact on soil microorgansms. The interaction between plant-root and rhizosphere microorganisms are reported to be a key factor for metal speciation in soils and could endorse a better efficiency in phytoremediation [64]. Earthworms secrete mucus which contains numerous plant-available nutrients and their casts were reported to be rich in amino acids, DOC and proteins [65]. These organic materials may chelate with Cd and thus promoting its absorption and transport [66]. 

**Figure 8:**
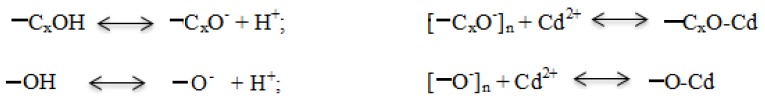
Possible mechanism of some different functional groups dissociating and complexing with Cd cations in soil.

The Cd concentration in both plants was significantly low inrice straw treatment. This could be explained on one hand by the low availability of Cd. Rice straw and earthworms interacted and changed the organic connections in soil by synthesizing humic compound which could significantly increase the number of functional groups capable of binding the heavy metal in soil (Figure 8). Indeed, there is a close correlation between soil microbial community as well as micro-fauna densities attached to plant residues. Soil microbial community and micro-fauna densities increased with the decomposition of organic matter present in soil. This could explain why the abundance of bacteria 16S rDNA and bacterial Shannon index significantly increased in rice straw either in a separate (S) or in combined (E + S) treatment (results not shown). The addition of rice straw in soil either with or without earthworms induces changes of certain functional groups which could become dissociated and complex with Cd cation.

## 5. Conclusions

The present study was carried out to explore how earthworms and rice straw influence Cd active forms in the soil. The results highlight that the cohabitation of earthworms, rice straw and plants could interactively enhance the decontamination of Cd contaminated-soil. The inoculation of earthworms in contaminated soil enhances Cd root uptake. Rice straw and earthworms interactively change the organic connections in soil, by increasing the number of functional groups capable of binding Cd in the soil. Our study offers clear and strong confirmation that both earthworms and rice straw could promote the reduction of Cd in soil and earthworms in particular enhance the phytoremediation of Cd by changing the soil properties and structure. These findings suggest that by the use of different ecological species of earthworms, rice straw and plants, it is possible to improve the remediation of Cd in polluted soil and will permit a better understanding of soil biological interaction effects on metal remediation technology. However, as the behavior of pollutants in natural contaminated environment could be changed due to the influence of several edaphic parameters, heavy metals interactions and so, further research with a mixture of metals is needed to establish the optimum use of this application. 

## Figures and Tables

**Table 1:** The body weight of *E. fetida* and *A. caliginosa* (mg) at the beginning and the end of the experiment. Low, medium and high represent respectively Cd concentration at 0.5; 1 and, 3 mg/kg Cd.

*Eisenia fetida* Body Weight (mg) *n* = 6					*Aporrectodea caliginosa* Body Weight (mg) *n* = 6			
	Initial Weight	Final Body Weight after 60 d			Initial Weight	Final Body Weight after 60 d		
Treat	0 mg.kg	Low	Medium	High	0 mg/kg	Low	Medium	High
**E_0_**	1835 ± 8.4 a	1832 ± 4.8 a	1841 ± 5.3 a	1830 ± 6.1 a	3044 ± 7.6 a	3049 ± 5.8 a	3039 ± 6.2 ab	3045 ± 6.5 a
**E_0_ + S**	1845 ± 6.8 ab	1883 ± 7.1 a	1856 ± 5.7 b	1853 ± 7.8 b	3025 ± 5.7 b	3043 ± 5.6 a	3038 ± 8.1 ab	3045 ± 5.9 a
**E_1_**	1825 ± 6.3 ab	1839 ± 5.7 a	1829 ± 6.3 ab	1827 ± 6.7 ab	3026 ± 4.8 ab	3028 ± 8.2 ab	3037 ± 7.6 a	3028 ± 4 ab
**E_1_ + S**	1834 ± 5.9 bc	1862 ± 4.5 a	1855 ± 5.4 b	1836 ± 5.7 bc	3032 ± 5.9 b	3045 ± 5.3 a	3048 ± 5.2 a	3039 ± 6.4 ab
**E_2_**	1842 ± 7.3 ab	1851 ± 4.8 a	1840 ± 7.6 ab	1845 ± 8.2 ab	3028 ± 7.3 ab	3037 ± 6.2 a	3027 ± 7.4 ab	3025 ± 7.1 ab
**E_2_ + S**	1859 ± 6.3 c	1871 ± 5.4 a	1864 ± 5.2 ab	1867 ± 5.1 ab	3041 ± 5.8 ab	3054 ± 6.5 a	3049 ± 5.7 a	3042 ± 8.3 ab

Data are the mean of three replicate ± SE and were compared by Tukey’s tests. The lowercase letters in the same raw (four values) indicates significant difference at *p* < 0.05. E_0_ indicates earthworms in no planted treatment, E_1_ and E_2_ indicate respectively earthworms in pot cropped with sunflower and oat.

**Table 2:** Influences of rice straw (S) and earthworms (E) on soil physical and chemical properties.

Treatments	CEC	% OM	Planted Pots		Unplanted Pots	
			pH	DOC	pH	DOC
**Ck**	12.83	10.23	7.4	38.356	7.28	30.569
**S**	11.58	37.24 ***	7.32	45.569 *	7.15	37.542 *
**E**	16.74 *	25.01 **	6.78 *	42.659 *	6.89 *	35.247 *
**E + S**	13.41	27.51 **	5.91 **	47.586 *	6.042 *	37.421 *

Data are the mean of three replicates tested by Two-way ANOVA analysis following by Tukey’s tests. The asterisks *, **, *** in the same column (four values) indicate significant difference at *p* < 0.05, *p* < 0.01 and 0.001 respectively. CEC indicates cation exchange capacity; OM, organic matter; DOC, dissolved organic carbon.

**Table 3:** Mean Cd content in earthworm tissues after 60 days of exposure.

*Eisenia fetida* (*n* = 6)				*Aporrectodea caliginosa* (*n* = 6)		
Treatment	Low	Medium	High	Low	Medium	High
E_0_	1.69 ± 0.3 bc	2.85 ± 0.4 c	5.55 ± 0.3 ab	2.15 ± 0.2 bc	4.29 ± 0.4 b	7.83 ± 0.3 b
E_0_ + S	2.52 ± 0.2 a	5.06 ± 0.6 a	7.73 ± 0.5 a	4.68 ± 0.4 a	6.37 ± 0.5 a	9.09 ± 0.8 a
E_1_	1.16 ± 0.4 c	2.35 ± 0.5 d	3.53 ± 0.6 c	1.95 ± 0.3 bc	2.74 ± 0.3 c	4.74 ± 0.4 d
E_1_ + S	1.97 ± 0.2 b	3.63 ± 0.4 b	5.72 ± 0.3 ab	2.69 ± 0.5 b	3.84 ± 0.7 bc	5.39 ± 0.6 c
E_2_	1.12 ± 0.3 c	2.47 ± 0.7 d	3.64 ± 0.6 c	1.85 ± 0.4 c	2.85 ± 0.3 c	3.95 ± 0.5 e
E_2_ + S	1.84 ± 0.4 b	2.97 ± 0.5 c	4.74 ± 0.4 b	2.58 ± 0.6 b	3.74 ± 0.5 bc	5.41 ± 0.6 c

Data are the mean of three replicate ± SE and were compared by Tukey’s tests. The lowercase letters in the same column (six values) indicates significant difference at *p* < 0.05. E_0_ indicates earthworms in no planted treatment, E_1_ and E_2_ indicate respectively earthworms in pot cropped with sunflower and oat plant.

**Table 4:** Bioconcentration factor (BCF), uptake rate (*k*_1_) and elimination rate (*k*_2_) constants in both earthworm species exposed to cadmium for 6 d.

Cd Concentration	*E. fetida*			*A. caliginosa*		
	BCF	*k*_1_ (d^−1^)	*k*_2_ (d^−1^)	BCF	*k*_1_ (d^−1^)	*k*_2_ (d^−1^)
**Low**	1.31 ± 0.03	0.024 ± 0.01	0.018 ± 0.06	1.72 ± 0.04	0.022 ± 0.02	0.013 ± 0.04
**Medium**	1.20 ± 0.01	0.05 ± 0.02	0.041 ± 0.04	1.38 ± 0.02	0.05 ± 0.03	0.019 ± 0.01
**High**	0.64 ± 1.01	0.03 ± 0.02	0.048 ± 0.07	0.83 ± 0.01	0.03 ± 0.01	0.021 ± 1.02

Data are the mean of three replicate ± SE and were compared by Duncan’s multiple range tests. BCF and bioaccumulation factor (BAF) were calculated as the ratio of the content of Cd in the earthworms to that in the soil. *k*_1_ was calculated by *k*_2_ × BCF.

**Table 5:** Bioconcentration factor (BCF) and translocation factor (TF) in both sunflower and oat plants after 60 days of exposure.

Sunflower Plant							Oat Plant					
	Low		Medium		High		Low		Medium		High	
Exp	BCF	TF	BCF	TF	BCF	TF	BCF	TF	BCF	TF	BCF	TF
**Ck**	1.21 ± 0.02	0.45 ± 0.04	0.65 ± 0.03	0.21 ± 0.02	0.35 ± 0.01	0.17 ± 0.04	1.67 ± 0.02	0.54 ± 0.03	1.12 ± 0.04	0.23 ± 0.02	0.59 ± 0.02	0.22 ± 0.01
**S**	0.64 ± 0.01	0.13 ± 0.02	0.38 ± 0.02	0.11 ± 0.03	0.31 ± 0.03	0.09 ± 0.03	0.78 ± 0.03	0.27 ± 0.02	0.53 ± 0.03	0.15 ± 0.03	0.37 ± 0.03	0.14 ± 0.03
**E**	1.38 ± 0.03	0.59 ± 0.03	0.82 ± 0.02	0.37 ± 0.01	0.67 ± 0.02	0.25 ± 0.02	1.43 ± 0.02	0.49 ± 0.04	0.88 ± 0.02	0.21 ± 0.03	0.43 ± 0.03	0.19 ± 0.02
**E + S**	1.01 ± 0.02	0.67 ± 0.04	0.46 ± 0.04	0.48 ± 0.03	0.39 ± 0.04	0.27 ± 0.01	1.21 ± 0.03	0.56 ± 0.02	0.66 ± 0.03	0.28 ± 0.02	0.40 ± 0.01	0.22 ± 0.03

Data are the mean of three replicate ± SD and were compared by Duncan’s multiple range tests. BCF was calculated as the ratio of the Cd content in the plant to that in the soil and TF as the ratio of the Cd content in the shoot to that of the root.

**Table 6:** Typical absorption bands and the main functional groups of rice straw.

Wavenumber (cm^−1^)	Functional Groups	Compounds
3708	O–H stretching	H_2_O
3430	O–H stretching	cellulose and lignin
2919 and 2853	C–H stretching vibration	Aliphatic materials
1640–1500	C=O	Ketone, carbonyl group
1450–1407	C=C stretching vibration	Aromatic skeletal
1388	C–H blending vibration	alkanes
1321–1302	C–O stretching and O–H blending	phenols, alcohols and esters
1242–1162	C–O–C stretching	aryl-alkyl ether
1070	C–O–C stretching vibration or C–O stretching and C–O deformation	ethanol group
1009	C–O–H and O–H blending	Decomposition of hemicellulose and cellulose
900–700	C–H	Aromatic hydrogen
700–400	C–C stretching	
